# Optimization of ZnO Nanorods Growth on Polyetheresulfone Electrospun Mats to Promote Antibacterial Properties

**DOI:** 10.3390/molecules25071696

**Published:** 2020-04-07

**Authors:** Mario Salmeri, Giulia Ognibene, Lorena Saitta, Cinzia Lombardo, Carlo Genovese, Matteo Barcellona, Alessandro D’Urso, Luca Spitaleri, Ignazio Blanco, Gianluca Cicala, Antonino Gulino, Maria Elena Fragalà

**Affiliations:** 1 Dipartimento di Scienze Biomediche e Biotecnologiche, Università degli Studi di Catania, Torre Biologica via S. Sofia, 97, 95123 Catania, Italy; msalmeri@unict.it (M.S.); cinzialombardo@hotmail.com (C.L.); carlo.genovese@studium.unict.it (C.G.); 2 Dipartimento di Ingegneria Civile ed Architettura (DICAR) and UdR-Catania Consorzio INSTM, Università degli Studi di Catania, Viale Andrea Doria 6, 95125 Catania, Italy; giuliaognibene@live.com (G.O.); lorena.saitta@phd.unict.it (L.S.); iblanco@unict.it (I.B.); gcicala@unict.it (G.C.); 3 Nacture S.r.l. - Spin-off Università degli Studi di Catania, 95123 Catania, Italy; 4 Dipartimento di Scienze Chimiche (DSC) and Consorzio INSTM UdR-Catania, Università degli Studi di Catania, Viale Andrea Doria, 6 95125 Catania, Italy; matteo.barcellona@studium.unict.it (M.B.); adurso@unict.it (A.D.); luca.spitaleri@phd.unict.it (L.S.); agulino@unict.it (A.G.)

**Keywords:** polyetheresulfone, ZnO, chemical bath deposition, electrospun mats, antibacterial action

## Abstract

Zinc oxide (ZnO) nanorods grown by chemical bath deposition (CBD) on the surface of polyetheresulfone (PES) electrospun fibers confer antimicrobial properties to the obtained hybrid inorganic–polymeric PES/ZnO mats. In particular, a decrement of bacteria colony forming units (CFU) is observed for both negative (*Escherichia coli*) and positive (*Staphylococcus aureus* and *Staphylococcus epidermidis*) Grams. Since antimicrobial action is strictly related to the quantity of ZnO present on surface, a CBD process optimization is performed to achieve the best results in terms of coverage uniformity and reproducibility. Scanning electron microscopy (SEM) and X-ray photoelectron spectroscopy (XPS) provide morphological and compositional analysis of PES/ZnO mats while thermogravimetric analysis (TGA) is useful to assess the best process conditions to guarantee the higher amount of ZnO with respect to PES scaffold. Biocidal action is associated to Zn^2+^ ion leaching in solution, easily indicated by UV–Vis measurement of metallation of free porphyrin layers deposited on glass.

## 1. Introduction

Electrospun nanofiber mats are currently investigated as engineered materials characterized by a high surface-to-volume ratio, high permeability, and porosity [1]. Electrospinning is a highly versatile technique to fabricate micro/nanofibers characterized by very high surface area and tailored mechanical properties [2]. A spinneret is connected to a syringe containing the polymer (melted or in solution) which is pumped in to form a drop on the needle of the syringe: the application of a potential difference between the needle and the collector determines the surface polarization of the drop and formation of electrical charges. When the electrostatic repulsion overexceeds surface tension, drop distortion causes formation of the Taylor’s cone and jet acceleration towards the collector. Solvent rapidly evaporates and fibers are deposited on the surface of the collector as non-woven fabric with random orientation. A wide selection of polymers can be used to produce fibrous scaffolds and surface functionalization or material doping tailor their composition for specific applications, spanning from biomedicine to environmental remediation [3,4,5,6,7]. In this perspective, polymeric nanofibers are often combined with other classes of materials—i.e., inorganic nanostructures and/or organic molecules—to achieve specific deliverable highlights. Combination of polymeric mats with semiconducting oxides, as zinc oxide and titanium oxide (ZnO, TiO2), improves mats performances by adding multifunctionalities such as photocatalytic properties and sensing actions [8,9].

Antibacterial properties of fibrous mats are currently under investigation [10,11,12,13] to provide innovative materials against bacteria surface contamination that causes severe infection and human health treats: in fact, microorganism colonies—responsible for biofilm formation—are developing an antibiotic resistance that makes this kind of contamination difficult to eradicate.

Both Gram-positive and Gram-negative bacteria are able to form biofilms [14], which represent the major causative agent of chronic and recurrent diseases [15,16]: in fact, it is estimated that more than 80% of human infections are biofilm-related [17,18]. In addition, the surfaces of commonly used prosthetic components—such as steel, titanium, and polymeric biomaterial—are susceptible to colonization of biofilm-forming bacterial species [19]. Antibiotics are often ineffective against biofilm-producing bacteria, due to their reduced growth rate and different gene expression [20,21]. It is well known that ZnO particles show antimicrobial activity against both Gram-positive and Gram-negative bacteria [22,23,24,25].

Recently, antimicrobial behavior of hybrid polymeric-inorganic materials is under investigation: however, literature refers to incorporation of ZnO inside the fibers by electrospinning of nanoparticles dispersion inside polymeric matrix [26], or to approaches based on combination of electrospraying and electrospinning processes dealing with presence of semi-exposed nanoparticles that improves biocidal action of mats [27].

We will focus on anti-biofilm ability of core–shell polyetheresulfones/zinc oxide (PES/ZnO) mats produced by using a bottom-up approach, during which ZnO nanorods are grown by Chemical Bath Deposition (CBD) from a nutrient solution on polyetheresulphone (PES) electrospun mats. CBD is a simple, versatile, and low-cost technique to grow ZnO nanostructures [28]. ZnO nanorods are formed on seeded PES fibers using a nutrient bath solution containing zinc acetate (as oxide metal precursor) and ethylendiamine (as complexant ligand) at 80 °C. Seeding and annealing (at T < 200 °C) steps are required to create nucleation centers on the fibers surface, aimed to promote homogeneous nucleation and growth of ZnO nanorods.

To note, PES is a thermoresistant polymer, insoluble in water and with outstanding hydrolytic stability in the 2–13 pH range, largely used as standard choice for water treatment membranes. Moreover, PES scaffolds are also used for wound healing application [29] PES shows Tgs higher than 190 °C and excellent thermal stability, thus representing an excellent choice for medium temperature (i.e., 100–140 °C) treatments as those reported herein for ZnO growth. All these properties are required to integrate PES electrospun nanofibers with the used CBD process. 

Herein, we compare and define the optimized ZnO deposition conditions to achieve high and uniform PES mats coverage and the compositional and morphological characterization of the material surface is associated to antimicrobial tests. In particular, PES/ZnO mats are tested for their anti-biofilm performances against *Staphylococcus aureus*, *Staphylococcus epidermidis*, and *Escherichia coli* bacterial strains. The obtained results demonstrate the ability of hybrid PES/ZnO mats to inhibit bacterial growth for both positive and negative Grams, thus confirming their potentialities as wound dressing materials.

## 2. Materials and Methods

Used PES grade, Veradel^®^ 3000P (Solvay, Bollate (MI) I-20021, Italy) was routinely used for water treatment in microfiltration and ultrafiltration membranes.

ZnO nanorods were grown onto the surface of the PES membranes by chemical bath deposition (CBD) process [30,31]. The first step of this process was a seeding procedure that consists of a membrane pre-treatment in zinc-acetate dihydrate (Zn(Ac)_2_ 2H_2_O) aqueous solution (0.5 M) (Figure S1). A proper support (Figure S2) is used to keep the sample at a constant height inside the seeding solution, where it remains immersed for 1 h under stirring at ambient temperature. After dipping, PES membrane was annealed by using different temperatures (<200 °C) and annealing times. During the second step, the supported seeded membrane is immersed in a 1:1 Zn(Ac)_2_ ·2H_2_O and ethylene diamine (EDA) solution (0.1 M), and kept magnetically stirred at 80 °C for a variable growth time, ranging from 30 min up to 3 h. The obtained mats were finally washed with distilled water and dried at 110 °C for 1 h.

Size and morphology of the ZnO nanorods were characterized by using a ZEISS SUPRA-55 VP (Oberkochen, Germany) field emission scanning electron microscope (SEM).

The electronic structure of the ZnO grown on PES mats was investigated by X-ray photoelectron spectra (XPS). XPS were measured at 45° take-off angle relative to the surface plane with a PHI 5600 Multi Technique System (Physical Electronics GmbH, Feldkirchen, Germany) (base pressure of the main chamber 1 × 10^−8^ Pa) [32,33]. Samples were excited with Al Kα X-ray radiation using pass energy of 5.85 eV. The instrumental energy resolution was ≤0.5 eV. Structures due to the Kα satellite radiations were subtracted from the spectra prior to data processing. The XPS peak intensities were obtained after Shirley background removal [32,33]. The atomic concentration analysis was performed by taking into account the relevant atomic sensitivity factors. Spectra calibration was achieved by fixing the C 1s signal at 285.0 eV [32,33].

Thermal degradations were performed in a Shimadzu DTG-60 simultaneous DTA-TG apparatus (Kyoto, Japan). Temperature, heat flow, and mass calibrations were fixed following the procedure suggested by Shimadzu and reported elsewhere [34]. Samples of about 3 mg, placed in a 40 μL alumina open pan, were heated, in static air atmosphere, at a heating rate of 10 °C⋅min^−1^ in the range of 25–800 °C. In order to correct the error in the mass determination, due to the reduction of the buoyancy force on increasing temperature, we used the blank method, recommended by the ICTAC Kinetics Committee. A thermogravimetric (TG) run with an empty pan (blank) was preliminarily performed in the same experimental conditions used for samples. The obtained blank curve was subtracted from the samples’ one, thus obtaining corrected degradation TG curves. At the end of each experiment, these data were used to plot the percentage of undegraded sample, (1−*D*)%, as a function of temperature, where *D* = (W_0_−W)/W_0_ and W_0_ and W are the masses at the starting point and during temperature scanning.

Anti-biofilm tests were performed on PES/ZnO materials: Table 1 reports the different strains used to check antimicrobial activity.

*Staphylococcus aureus* ATCC 29,213 and *Escherichia coli* ATCC 35218 were purchased from the American Type Culture Collection (Rockville, MD, USA) and used as reference strains. Tests were performed according to Clinical and Laboratory Standards Institute guidelines [35]. Isolated colonies on Mueller-Hinton agar plates were suspended in 0.85% NaCl, to achieve a turbidity equivalent to 1 McFarland Standard (3.0 × 10^8^ CFU/mL). The turbidity evaluation was carried out by spectrophotometric reading at λ = 600 nm (OD600) (Synergy HT – Biotech, Winooski, VT, USA). After a dilution in the 1:100 ratio in Trypticase Soy Broth (TSB) (Oxoid, Thermo Fisher Scientific, Waltham, MA, USA), the bacterial suspensions were inoculated in a 12-well cell culture plate containing PES/ZnO to a final concentration of 3.0 × 10^6^ CFU/mL. The microplates were incubated for 72 h and TSB was changed every 24 h. After 72 h, the medium was aspirated and the wells were washed twice with a phosphate buffered saline (PBS) solution to remove planktonic cells. The microplates were rinsed with 1 mL of PBS and vortexed for two minutes at 200 rpm. The total bacterial count in the biofilms was determined by the plate count method. Dilutions in the 1:10 ratio of the cells released from the material were made and plated into Luria-Bertani agar (Oxoid) to determine the CFU/mL. A microplate containing PES and 1 mL per well of TSB was used as negative control. Results are expressed as mean of three experiments.

UV–Vis spectroscopy (Jasco V-730 UV-Visible Spectrophotometers, Easton, MD, USA) was used to measure Zn^2+^ ion leaching, by exploiting the porphyrin ability to complex heavy metal ions. In particular, glass slides were dipped in a 5 μM aqueous solution of 5,10,15,20-tetrakis(N-methylpyridinium-4-yl)porphyrin (H2T4) to deposit a thin porphyrin layer. PES/ZnO mats were dipped in DI water (at neutral pH) for 24 or 48 h and functionalized glass slides are able to detect the release of Zn^2+^, due to the metal complexation in the macrocycle cavity, easily detectable by the modification of the UV–Vis glass spectra [36,37].

## 3. Results

### 3.1. ZnO Growth Optimization

**Figure 1:**
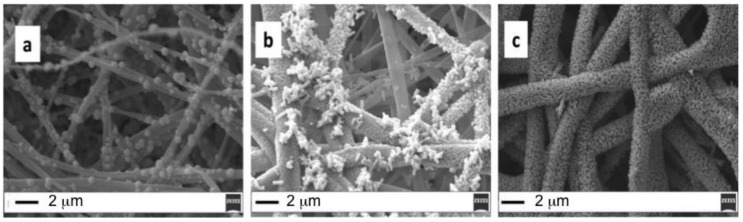
(**a**) Seed 1 h, annealing 150 °C 1 h, CBD 1 h; (**b**) Seed 1 h, annealing 150 °C 1 h, CBD 2 hs; (**c**) Seed 1 h, annealing 150 °C 1 h, CBD 3 h (scale bar 2 μm).

ZnO CBD process conditions were changed to optimize the deposition in terms of both core–shell fibers morphology and uniformity. In Figure 1 we can clearly observe how CBD growth time plays an important role in increasing both core–shell fiber diameters and homogeneity.

**Figure 2:**
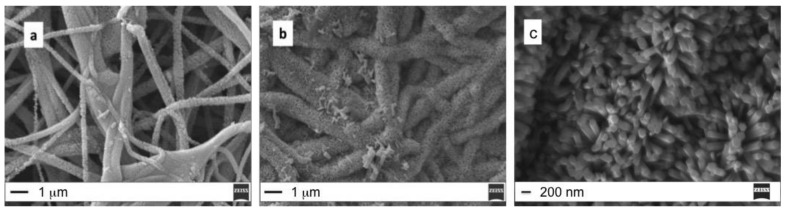
(**a**) Seed 1h, annealing 180 °C 1 h, CBD 30 min; (**b**) Seed 1 h, annealing 180 °C 1 h, CBD 1 h (scale bar 1 μm); (**c**) SEM high magnification image of ZnO nanorods (scale bar 200 nm).

In particular, by keeping constant the seeding conditions (1 h dipping in Zn(Ac)_2_ solution and 1 h annealing at 150 °C), the CBD time is varied from 1 h (Figure 1a) to 2 h (Figure 1b) up to 3 h (Figure 1c). Notably, the ZnO fiber average diameter increased from 500 nm up to 2 micron, when the growth time is increased from 1 to 3 h.

Setting a CBD growth time of 1 h, the formation of isolate ZnO aggregates is visible on the top of the PES fiber surface, but the mean diameter is about 500 nm. The presence of larger fibers (mean diameter > 1 μm), coated with ZnO nanorods creating a brush-like shell, is evident in Figure 1b, where, however, uncovered fibers are still visible. More uniform fiber coverage is achieved by prolonging the CBD growth time up to 3 h (Figure 1c). To note, the growth of ZnO nanorods increases the fiber average diameter (from 500 nm up to 2 micron). From the above observations, it emerges that the annealing temperature plays an important role on the growth of the ZnO nanorods since ZnO crystallites are firstly required for ZnO nanorod growth [8]. In order to define the role of the seeding conditions for fibers coating, the annealing temperature is raised up to 180 °C (Figure 2): in this case the CBD growth time can be reduced to 30 min (Figure 2a) or 1 h (Figure 2b) to obtain a significant coverage with ZnO nanostructures (Figure 2c).

These results confirm that the increase of the annealing temperature is useful to reduce the CBD growth time.

ZnO coverage uniformity, estimated by SEM images is confirmed by TGA analysis of two different PES/ZnO mats obtained by using two different growth conditions (Table 2).

**Figure 3:**
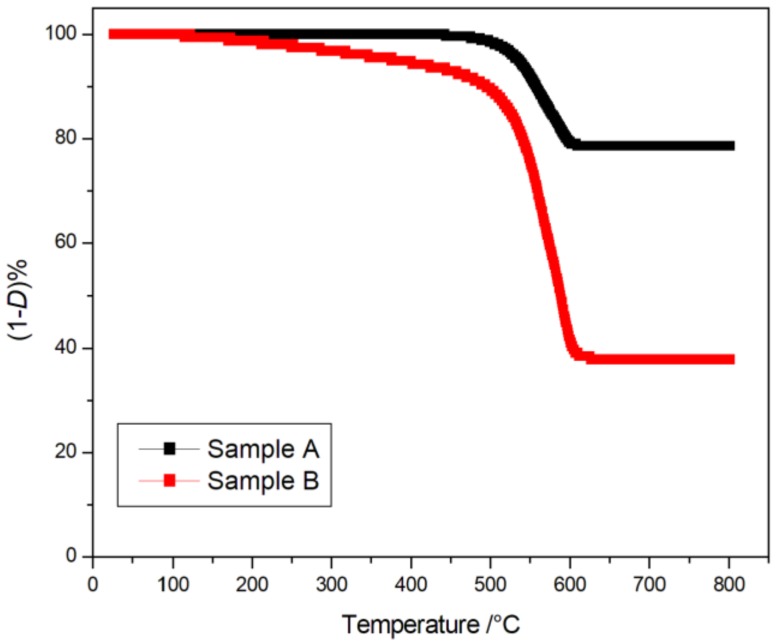
TG degradation curves in air for sample A and sample B (ramp rate: 10 °C·min^−1^).

Samples are analyzed in three different regions, at the edges and at the center of a rectangular woven fabric and the weight loss during the temperature scanning related to the PES degradation is evaluated. Figure 3 shows the results of the TGA analyses for both A and B samples.

In particular, for sample A the degradation starts at 532 °C (Temperature at 5% of degradation, *T*_5%_) and ends at ~600 °C following a one step pathway. A similar trend, but starting at lower temperature (*T*_5%_ = 376.8 °C), is obtained for sample B. Such behavior can be attributed to the presence of the ZnO that improves the thermal resistance of the polymer by retarding its degradation process. In fact, the high thermal capacity of the inorganic oxide can retard the polymer thermal degradation. The residual weight percentage ((1−*D*)%) calculated for sample A (about 80%) is higher than that obtained for sample B (about 60%) due to a more uniform coverage with ZnO nanostructures. Noteworthy, in absence of ZnO, the expected weight loss (*D*%) due to the PES degradation was about 70 [8].

The obtained mean *D*% values, calculated by analyzing three different areas of PES/ZnO mats, the standard deviation (StDev) and mean square errors (SE Mean) are reported in Table 3.

**Figure 4:**
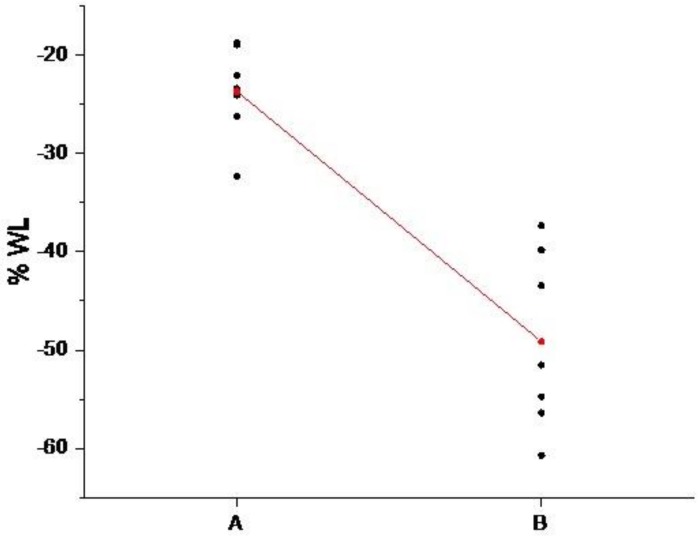
Individual value plot of the % weight loss of sample A and sample B.

A pooled *t*-test is carried out by using the software Minitab17^®^: two different conditions (30 and 60 min) of the growth time factor are compared considering seven replications. Evaluated means are significantly different (*p* < 0.05) from each other (Figure 4) and this result remarks the importance of growth time on the coverage uniformity.

#### XPS Characterization

**Figure 5:**
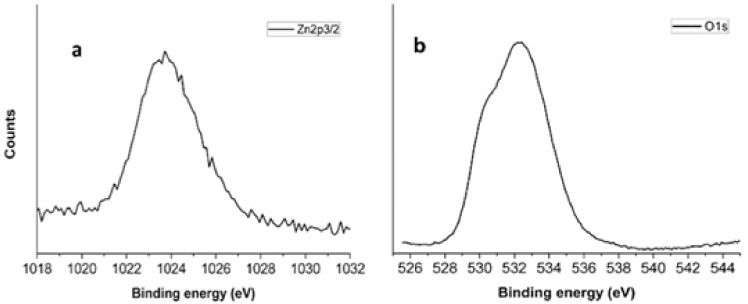
Zn 2p_3/2_ (**a**) and O1s (**b**) XPS peaks related to ZnO grown on PES mats by CBD.

XPS analysis confirms the ZnO presence in the external shell and Figure 5a shows the Zn 2p_3/2_ (1024.0 eV) spin-orbit component, typically associated to Zn^2+^ ions in ZnO [38,39,40,41]. The oxygen 1s peak (Figure 5b) is constituted by two main components: the component at lower binding energy is associated to Zn-O states (530.2 eV) [42,43], while the other at higher binding energy (533.2 eV) is due to presence of hydroxyl groups onto the sample surface [32,33].

### 3.2. Antimicrobial Test

The biofilm represents a matrix composed by extracellular polymeric substances (EPS). The biofilm formation process can be described by five different stages: the reversible bacterial adhesion to the surface; the conversion of reversible adhesion to irreversible adhesion; the formation of the biofilm; the maturation of the biofilm; the degeneration of the biofilm; and return of bacteria to planktonic state. The biofilm allows bacterial cells to adapt against adverse environmental conditions and to increase tolerance to antibiotics [44]. Anti-biofilm properties of ZnO/PES are investigated against *Escherichia coli*, *Staphylococcus epidermidis* and *Staphylococcus aureus.*

**Figure 6:**
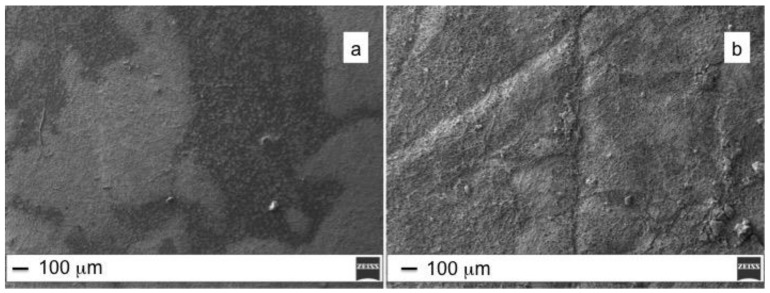
PES (**a**) and PES/ZnO (**b**) mats treated with *E. coli* ATCC 35,218 (Scale bar 100 μm).

As to biofilm formation, in Figure 6 is reported an example obtained in presence of Gram-negative bacteria reunited in small clumps across the electrospun membrane surface. However, each bacterial strain exhibits different ability to develop biofilms.

Generally, bacterial adherence on surface depends on many parameters related to surface morphology and composition. The hydrophobicity of the bacterial cell surface typically determines the affinity towards hydrophobic surface of polymeric mats. Roughness, on the other hand, promotes bacterial adhesion and biofilm formation due to the increased surface area and presence of favorable sites for colonization (i.e., wrinkles, depression, edges). *E. coli* ATCC 35218 strain forms a thick biofilm on the top of PES fibers, well visible in the SEM image (Figure 6a) as dark regions that completely cover the polymeric fibers. On the contrary, the PES/ZnO surface, despite the higher roughness related to formation of ZnO nanorods, appeared less colonized (Figure 6b).

The analysis of the results indicates an anti-biofilm activity of the coating ZnO nanorods, whereby fewer bacteria are attached on the membranes, as summarized in Table 4 reporting the optical density at λ = 600 nm (OD_600_) of the biofilm-detached cells. OD_600_ provides a measure of the light scattered, which manifests itself as absorbance. Colony counts of adherent bacteria, expressed as colony-forming unit/mL (CFU/mL), reveal a 1-log reduction in viable bacteria recovered from the membrane surface for S. aureus 004/392 (1.12 × 10^9^ CFU/mL for PES compared with 6.84 × 10^8^ CFU/mL for PES/ZnO). A similar reduction is observed for *Escherichia coli* ATCC 35218 Gram-negative strain (1.28 × 10^9^ CFU/mL for PES compared with 7.84 × 10^8^ for PES/ZnO). For the remaining bacterial strains, although to a lesser extent, PES/ZnO mats are able to reduce recoverable CFUs as compared with the biofilm from the ZnO-free control.

**Figure 7:**
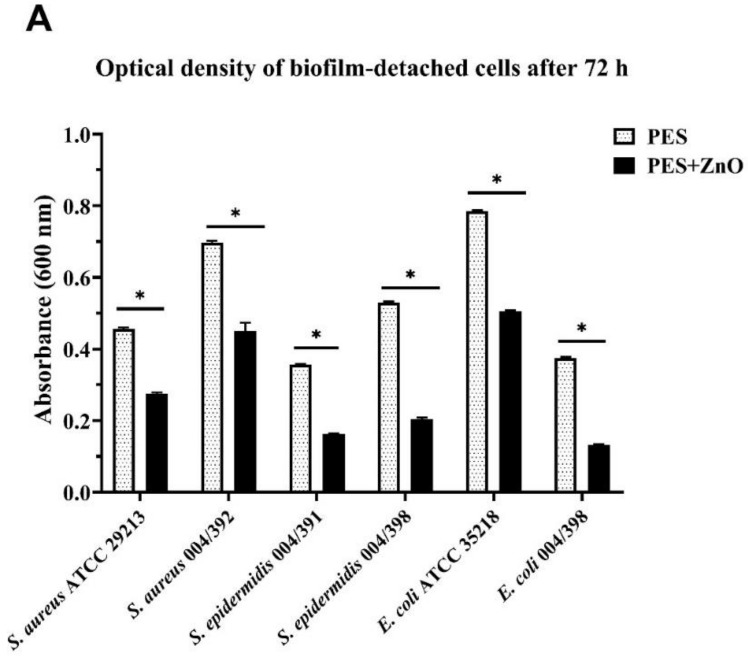

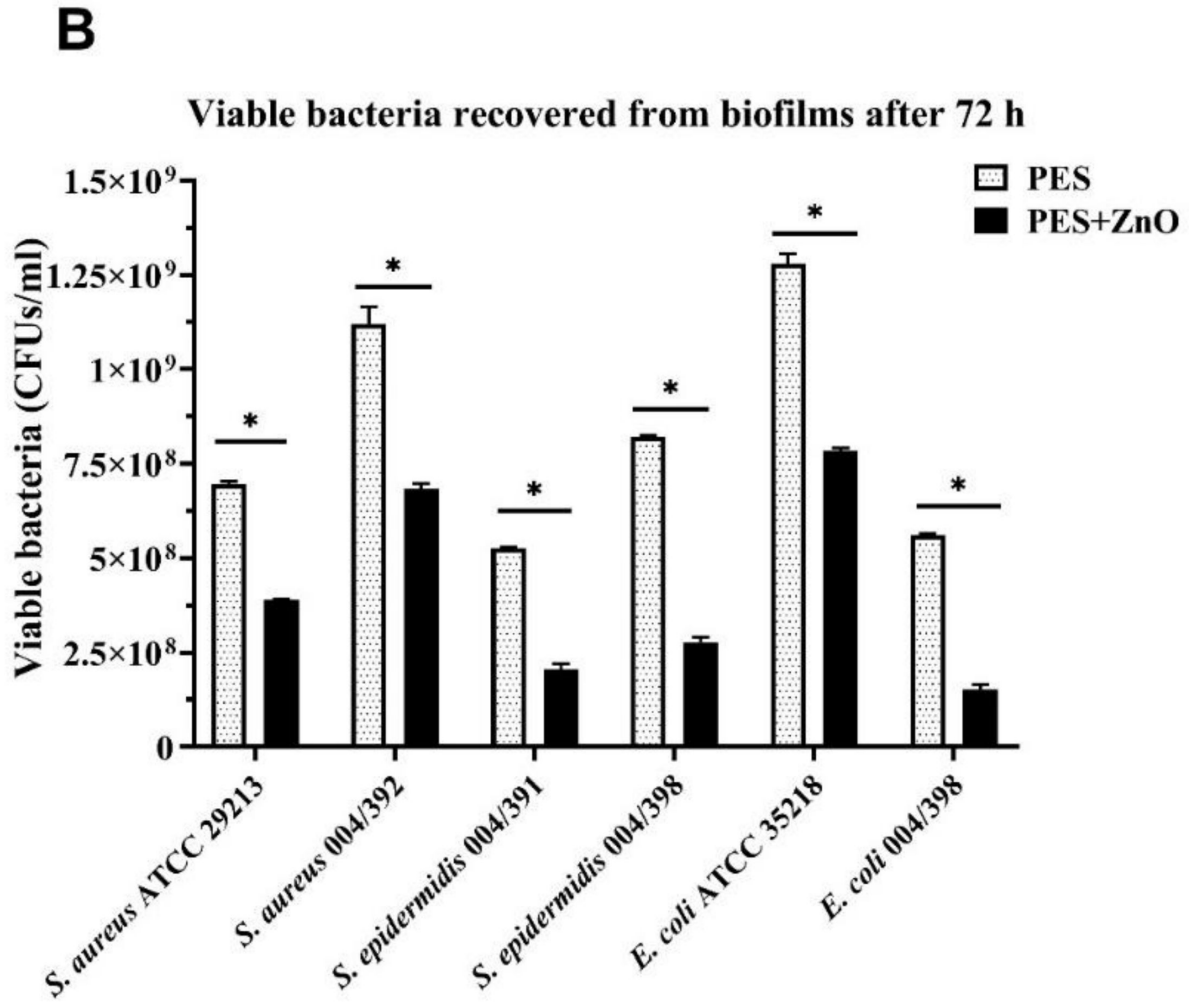
Effect of ZnO addition to PES on biofilm formation. Bacterial strains were grown on PES both in absence or presence of zinc oxide for 72 h. The histograms represent the OD_600_ values of biofilm detached cells (**A**) and viable bacteria (CFUs/mL) recovered from biofilms (**B**) for each bacterial strain, in the two experimental conditions. The bars represent the means ± SD of three independent experiments performed in triplicate (S.D. = standard deviation). Statistically significant differences, determined by two-way analysis of variance ANOVA, are indicated by *p < 0.0001 PES/ZnO vs. PES.

The two-way analysis of variance ANOVA indicates a statistically significant reduction in viable bacteria, with a *p*-value < 0.0001 (Figure 7).

## 4. Discussion

The observed antimicrobial effect of PES/ZnO mats can be rationalized by two factors: i) morphological modification of fibrous mats; and ii) Zn^2+^ release. In particular, ZnO growth was responsible for the increased diameter of fibers and, the brush-like architecture of the resulting core–shell nanostructure can be responsible for a different interfacial interaction between bacteria and the investigated surfaces [8,9].

**Figure 8:**
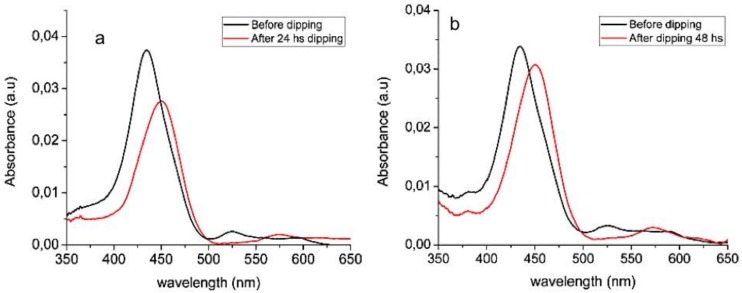
UV–Vis spectra of glass slides functionalized with H2T4 before (black line) and after (red line) immersion in DI water containing Zn^2+^ ions released by ZnO/PES mats dipped in water for (**a**) 24 h and (**b**) 48 h.

In fact, ZnO nanostructures are considered antimicrobial material towards prokaryotic and eukaryotic systems and Zn^2+^ ions leaching is assumed to be responsible for the observed cytotoxicity [45,46]. The mechanism for antimicrobial effect is not well known, although it could be due to the binding of ZnO particles on the bacterial surface through electrostatic forces [47], disruption of cell membrane with loss of its functionality [48], and generation of hydrogen peroxide [49]. It was demonstrated that the ZnO nanoparticles structure, chemical composition and morphology play a crucial role in antimicrobial activity [50]: Zn^2+^ cations, released from ZnO nanorods dissolution in water, are able to interact with different bacterial target sites [51,52]. The higher surface area of nanostructures with respect to that of bulk materials can promote a faster ion release in water or physiological solutions. To demonstrate the Zn^2+^ leaching from PES/ZnO mats we use the ability of porphyrins to coordinate metal ions inside their cavity, process associated to significant modification of the porphyrin UV–Vis spectrum [36,37]. In particular, herein, porphyrin metallation resulting from Zn^2+^ release caused by mats dipping in distillated water (Figure 8) is detected by following the spectrophotometric UV–Vis signal evolution of glass slides non-covalently functionalized with a cationic porphyrin (H2T4) layer.

More in detail, glass slides are dipped in a cationic H2T4 solution to promote the spontaneous adhesion of a porphyrin layer, well traceable by UV–Vis measurement of glass whose spectrum shows an intense Soret band at 436 nm (Figure 8, black line). When the free porphyrin cavity is complexed by metal ions, a red shift of Soret band from 436 nm to 452 nm, and spectral modification of the Q-band region (spectral range 500–650 nm) are associated to the formation of the ZnT4 complex (Figure 8, red line). The amount of Zn^2+^ ions in solution increases upon prolonging the fibers dipping time from 24 (Figure 8a) to 48 h (Figure 8b), as confirmed by the increase of the new Soret band intensity. Accordingly, mats’ dipping in culture broth containing bacteria cells causes Zn^2+^ ions release with a consequent cytotoxic effect.

## 5. Conclusions

Our results suggest that ZnO nanorods coatings for PES electrospun nanofibers could be used for biomedical applications prone to excessive bacterial growth (such us patches or prosthesis for damage to the skin).

ZnO/PES mats demonstrate activity towards both positive and negative grams, confirmed by a general reduction of colony counts of adherent bacteria (CFU). Zn^2+^ ions release, verified by spectrophotometric investigation of the free porphyrin (H2T4) layer complexation, is assumed to be responsible for the biocidal action.

The proposed strategy to couple polymeric electrospun mats with ZnO has the advantage, with respect to other approaches based on incorporation of the ZnO semiconducting oxide insides the fibers, to directly expose bacteria to the oxide. Optimization of the process conditions for chemical bath deposition of ZnO nanorods, to fabricate core–shell PES/ZnO fibers aimed to overcome limitations related to control antimicrobial compound [53]. To note, ZnO has a high isoelectric point (IEP > 9), thus its surface is negatively charged in physiological conditions, while wall cell structure is more complex and it is not simple to determine the overall surface charge. Bacteria are more prone to adhere on positively charged surface, but surface growth is generally observed on negatively charged surfaces [54]. In the present case, we have demonstrated how the presence of ZnO nanostructures on the mats exposed surface leads to a more extensive Zn^2+^ ions release exerting a cytotoxic action and causing a breakage of bacteria cell integrity. Further studies are on-going to study the anti-biofilm mechanism of action and the effective Zn^2+^ cytotoxic dose for cell lines.

## Figures and Tables

**Table 1:** Gram positive and negative strains used to test PES/ZnO membranes. Strain numbers refer to an internal directory for bacteria.

Bacterial Strains	Source
Gram-positive	
*Staphylococcus aureus* ATCC 29213	Standard
*Staphylococcus aureus* 004/392	Septicemia
*Staphylococcus epidermidis* 004/391	Endovascular catheter-associated infection
*Staphylococcus epidermidis* 004/398	Endovascular catheter-associated infection
Gram-negative	
*Escherichia coli* ATCC 35218	Standard
*Escherichia coli* 004/398	Peritonitis

**Table 2:** Processing conditions used for PES/ZnO mats to study ZnO coverage uniformity by TGA.

Sample	Seed Annealing Time (min)	Annealing (°C)	CBD Growth Time (min)
A	60	180	60
B	60	180	30

**Table 3:** TGA weight loss for A and B samples: mean, standard deviation, and mean square error.

Sample	Mean *D*%	StDev	SE Mean
A	−23.66	4.68	3.4
B	−49.14	8.97	1.8

**Table 4:** Optical density (OD600) values and total bacterial count (CFUs/mL) obtained for PES and PES/ZnO fibers inoculated with different bacterial strains. Results are expressed as mean of three experiments ± standard deviation.

Bacterial Strains	PES		PES/ZnO	
**Gram-positive**	**OD_600_**	**CFUs/mL**	**OD_600_**	**CFUs/mL**
*S. aureus* ATCC 29213	0.460 ± 0.003	6.96 × 10^8^ ± 7.02 × 10^6^	0.280 ± 0.004	3.89 ×10^8^ ± 3.51 ×10^6^
*S. aureus* 004/392	0.700 ± 0.005	1.12 × 10^9^ ± 4.58 ×10^7^	0.450 ± 0.024	6.84 ×10^8^ ± 1.45 ×10^7^
*S. epidermidis* 004/391	0.360 ± 0.003	5.26 × 10^8^ ± 3.61 ×10^6^	0.160 ± 0.003	2.04 ×10^8^ ± 1.64 ×10^7^
*S. epidermidis* 004/398	0.530 ± 0.003	8.22 × 10^8^ ± 3.00 ×10^6^	0.200 ± 0.005	2.78 ×10^8^ ± 1.35 ×10^7^
**Gram-negative**	**OD_600_**	**CFUs/mL**	**OD_600_**	**CFUs/mL**
*E. coli* ATCC 35218	0.790 ± 0.002	1.28 ×10^9^ ± 2.65 ×10^7^	0.510 ± 0.003	7.84 ×10^8^ ± 7.64 ×10^6^
*E. coli* 004/398	0.380 ± 0.003	5.61 ×10^8^ ± 4.00 ×10^6^	0.130 ± 0.003	1.53 ×10^8^ ± 1.23 ×10^7^

## Supplementary Materials

The following are available online at www.mdpi.com/xxx/s1,s2. Figure S1. Supported PES mat immersed in seeding bath; Figure S2. Ultem 9085^TM^ support rendering.

## Abbreviations

Polyetheresulphone (PES)
Chemical bath deposition (CBD)
Zinc oxide (ZnO)
Titanium oxide (TiO_2_)
Zinc acetate dihydrate (Zn(Ac)_2_ ·2H2O)
Thermogravimetric analysis (TGA)
X-ray photoelectron spectroscopy (XPS)
Scanning electron microscopy (SEM)
Tetracationic tetrakis (N-methylpyridinium-4-yl)porphyrin (H2T4)
Phosphate buffered saline (PBS)
Colony-forming unit/mL (CFU/mL)
