# Supplementary material for: Optimization of ZnO Nanorods Growth on Polyetheresulfone Electrospun Mats to Promote Antibacterial Properties
Source: Molecules. 2020 Apr 7;25(7):1696. doi: 10.3390/molecules25071696 (PMC7180436; doi:10.3390/molecules25071696)
Supplement: Supplementary file 1 [file molecules-25-01696-s001.pdf]

molecules

Article

Optimization of ZnO Nanorods Growth on Polyetheresulfone Electrospun Mats to Promote Antibacterial Properties

Mario Salmeri [1], Giulia Ognibene [2], Lorena Saitta [2], Cinzia Lombardo [1], Carlo Genovese [1,3], Matteo Barcellona [4], Alessandro D'Urso [4], Luca Spitaleri [4], Ignazio Blanco [2], Gianluca Cicala [2], Antonino Gulino [4] and Maria Elena Fragalà [4,*]

[1] Dipartimento di Scienze Biomediche e Biotecnologiche, Università degli Studi di Catania, Torre Biologica via S. Sofia, 97, 95123 Catania, Italy; msalmeri@unict.it (M.S.); cinzialombardo@hotmail.com (C.L.); carlo.genovese@studium.unict.it (C.G.)

[2] Dipartimento di Ingegneria Civile ed Architettura (DICAR) and UdR-Catania Consorzio INSTM, Università degli Studi di Catania, Viale Andrea Doria 6, 95125 Catania, Italy; giuliaognibene@live.com (G.O.); lorena.saitta@phd.unict.it (L.S.); iblanco@unict.it (I.B.); gcicala@unict.it (G.C.);

[3] Nacture S.r.l. - Spin-off Università degli Studi di Catania; 95123 Catania, Italy

[4] Dipartimento di Scienze Chimiche (DSC) and Consorzio INSTM UdR-Catania, Università degli Studi di Catania, Viale Andrea Doria, 6 95125 Catania; matteo.barcellona@studium.unict.it (M.B.); adurso@unict.it (A.D.); luca.spitaleri@phd.unict.it (L.S.); agulino@unict.it (A.G.); me.fragala@unict.it. (M.E.F)

* Correspondence: me.fragala@unict.it; Tel.: +39-095-7385149

Academic Editors: Keith M. Forward and Jiashen Li

ZnO Chemical Bath Deposition on PES electrospun mats

3D printed support was used both for mats seeding and CBD procedures, as shown in Figure S1.

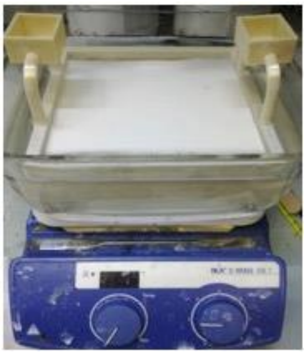

Figure S1. Supported PES mat immersed in seeding bath.

The support was made by using a high temperature thermoplastic blend consisting of Polyetherimide (PEI) with a Polycarbonate copolymer (Ultem 9085™ -Tan grade) and it was designed by Solidworks® (Figure S2). It was realized by using Fortus 3D printer (Fortus 400mc-Stratasys, Los Angeles, USA) and printing volume was 355 x 254 x 254 mm.

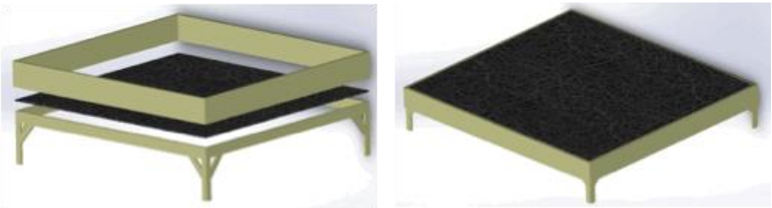

Figure S2. Ultem 9085™ support rendering.
